# A draft genome sequence of *Pseudomonas veronii* R4: a grapevine (*Vitis vinifera* L.) root-associated strain with high biocontrol potential

**DOI:** 10.1186/s40793-016-0198-y

**Published:** 2016-10-11

**Authors:** Christian Montes, Fabiola Altimira, Hayron Canchignia, Álvaro Castro, Evelyn Sánchez, María Miccono, Eduardo Tapia, Álvaro Sequeida, Jorge Valdés, Paz Tapia, Carolina González, Humberto Prieto

**Affiliations:** 1 Biotechnology Doctoral Program, Universidad Técnica Federico Santa María-Pontificia Universidad Católica de Valparaíso, Valparaíso, Chile; 2 Universidad Técnica Estatal de Quevedo, Facultad de Ciencias Agrarias, Av. Quito Km 1.5 road, Santo Domingo de los Tsachilas, Quevedo, Los Ríos Ecuador 120501; 3 Fraunhofer Chile Research Foundation, Av. Mariano Sánchez Fontecilla 310, 14th Floor, Las Condes Santiago, Chile 7550296; 4 Biotechnology Laboratory, La Platina Research Station, Instituto de Investigaciones Agropecuarias, Santa Rosa 11610, La Pintana, Santiago, 8831314 Chile

**Keywords:** *Pseudomonas veronii*, *Pseudomonas* spp., *Xiphinema index*, *Vitis vinifera* L, Exoproteases, Exolipases, Biocontrol

## Abstract

A new plant commensal *Pseudomonas veronii* isolate (strain R4) was identified from a *Xiphinema index* biocontrol screen. Isolated from grapevine roots from vineyards in central Chile, the strain R4 exhibited a slower yet equivalently effective nematicide activity as the well-characterized *P. protegens* CHA0. Whole genome sequencing of strain R4 and comparative analysis among the available *Pseudomonas* spp. genomes allowed for the identification of gene clusters that encode putative extracellular proteases and lipase synthesis and secretion systems, which are proposed to mediate—at least in part—the observed nematicidal activity. In addition, R4 strain presented relevant gene clusters related to metal tolerance, which is typical in *P. veronii*. Bioinformatics analyses also showed gene clusters associated with plant growth promoting activity, such as indole-3-acetic acid synthesis. In addition, the strain R4 genome presented a metabolic gene clusters associated with phosphate and ammonia biotransformation from soil, which could improve their availability for plants.

**Electronic supplementary material:**

The online version of this article (doi:10.1186/s40793-016-0198-y) contains supplementary material, which is available to authorized users.

## Introduction

Wine and table grape cultivar productions strongly depend on plant root health and physiology. Soil-borne pathogens affecting these systems avoid water and nutrients uptake and lead to several physiological disorders such as root rot and blackening or plant wilt and stunting. In Chile, several genera of plant-parasitic nematodes are limiting factors for grape production, and one of the most damaging is the dagger *Xiphinema index* [1]. This nematode is also the natural vector of the *Grape fan leaf virus*, a widespread disease that affects important grape productive areas of the country [2, 3].

*Pseudomonas* spp. belonging to the *fluorescens* group are recognized ubiquitous soil nematicidal agents that can also promote plant health [4, 5]. Among *Pseudomonas* sp. strains exhibiting antagonistic activity against nematodes of agronomic relevance, the *P. protegens* strain CHA0 [6] has shown an extraordinary capacity against the root-knot nematodes *Meloidogyne javanica* and *M. incognita* by producing exoproteases 2,4-DAPG and HCN [7]. The latter was also described to mediate the nematicidal activity of *P. chlororaphis* O6 over *Meloidogyne hapla* [8], a broad host-spectrum plant nematode. Nematicidal repertoires in pseudomonads obey to an important degree of genome heterogeneity within the species group; whereas comparison of 16S ribosomal RNA (rRNA) gene sequences have shown a defined clustering for *P. protegens* strains, the use of antimicrobial secondary metabolites has led to wrong classification in *P. fluorescens* and *P. chlororaphis* [9]. In addition, whole genome sequence data from different *P. fluorescens* strains have highlighted a strain-to-strain variation and diversity [10]; whereas a conserved set of genes forming a core genome represents only 45–52 % of the genome of any individual strain, important variable regions and several hundred genes are unique to each genome [10].

**Fig. 1:**
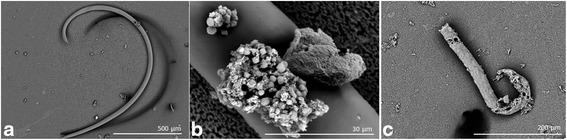
Nematicidal activity of the *P. veronii* strain R4 over *Xiphinema index* individuals. Nematicidal activity of strain R4 cell supernatants was obtained from cell cultures grown with milk induction [12], pelleted with acetone, and resuspended using phosphate buffer. Total proteins (20 μg) were added to the wells of 96-well microplates containing 100 μL of buffer and 30 nematodes. The plates were incubated at 24 ± 1 °C for 3 h, and the samples were analysed using scanning electron microscopy (SEM). Initial cuticle degradation in *X. index* individuals appeared on discrete areas of nematodes’ bodies (**a** and **b**), which after challenge led to whole degradation (**c**)

Currently, biocontrol activity has not been described for *P. veronii* isolates, which have been largely renowned by their biosorption/bioremediation capabilities [11]. In the present work, we report the whole genome sequencing and characterization of a new *P. veronii* strain R4. This isolate was first identified from a *X. index* biocontrol panel and presents a highly effective nematicide activity as compared with *P. protegens* CHA0. Primarily, the strain R4 cell supernatants resulted in important nematode disruption (Fig. 1 a-c), and candidate proteins responsible for this activity have been isolated, partially sequenced, and identified in these extracts [12].

## Organism information

### Classification and features

**Fig. 2:**
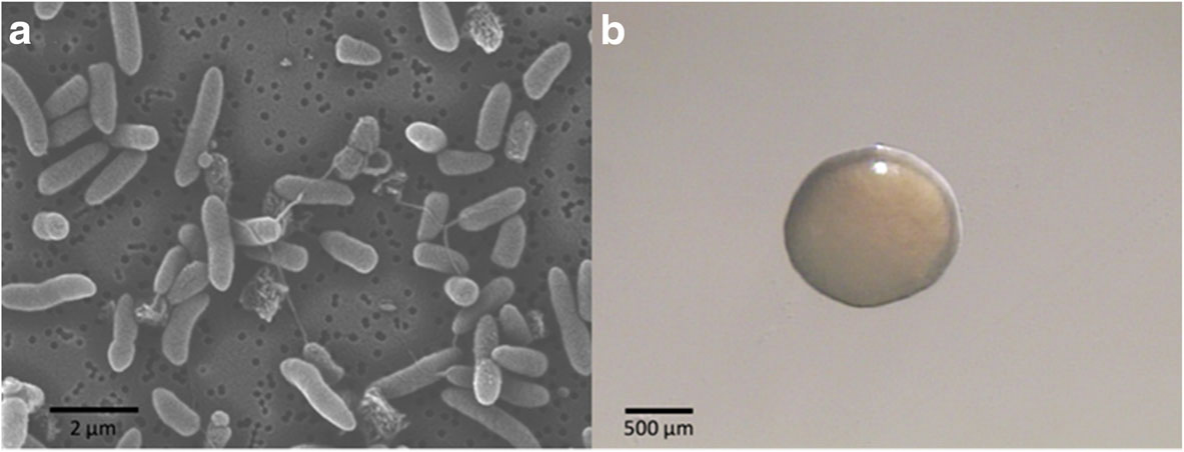
Microscopy analyses of the *P. veronii* strain R4. Images recording the morphological aspect of the strain R4 cells (**a**) or an individual colony (**b**) were acquired using SEM and a light microscope, respectively. Images were acquired to samples grown for 24 h in KB agar medium at 28 °C

**Fig. 3:**
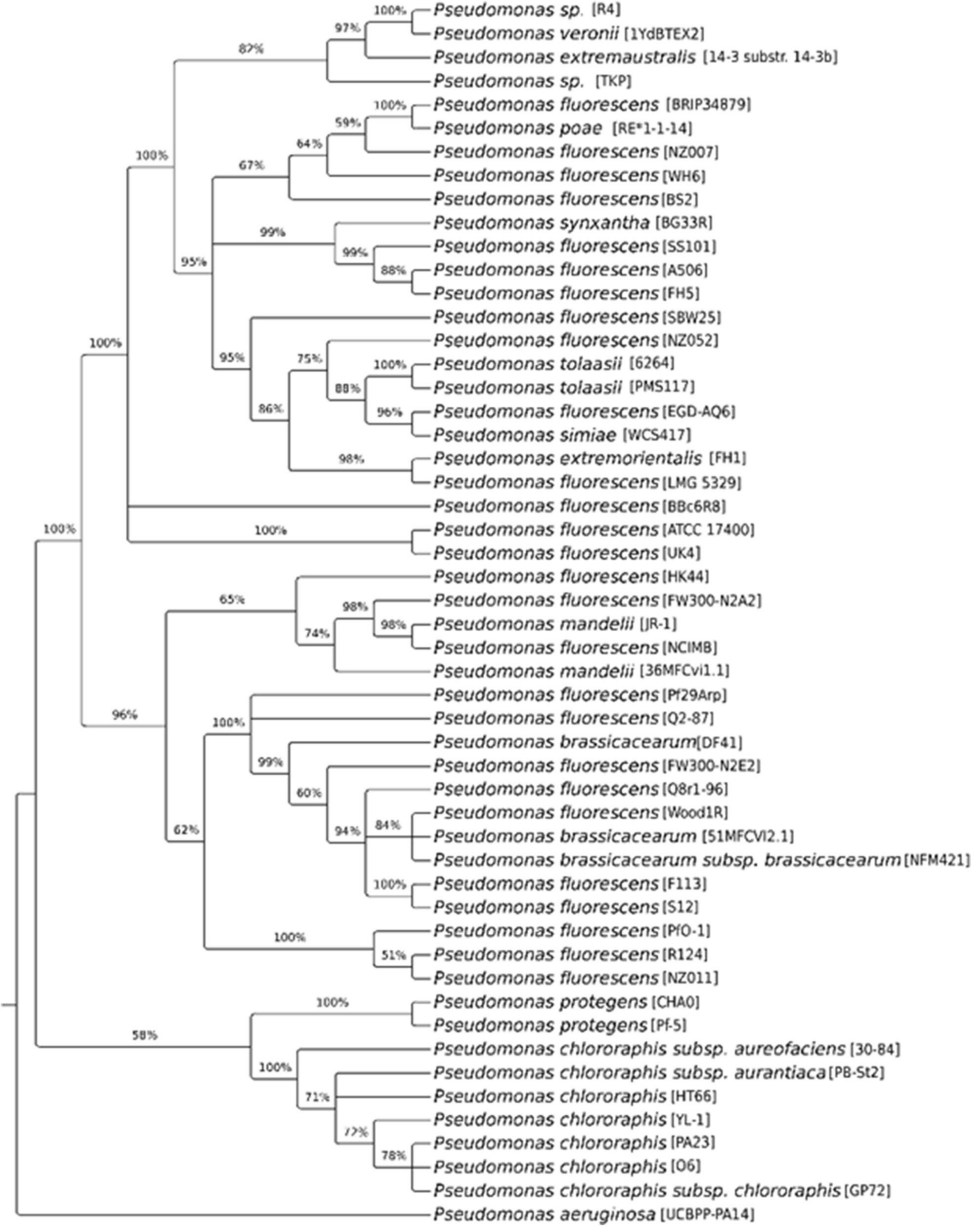
Concatenated alignments of the 31 highly conserved COGs for the 50 members of the *Pseudomonas fluorescens* group described by [31], which presents a sequenced genome (draft or final) and R4 strain were made with MUSCLE [47]. The poorly aligned and divergent regions were eliminated using Gblocks [48], as described by [49]. The phylogenetic tree was reconstructed using the maximum likelihood method implemented in the PhyML v3.0 program [50] using the Dayhoff substitution model. Reliability for internal branches was assessed using the Shimodaira-Hasegawa-like Approximate-Likelihood [51]. The resulting tree was visualized using TreeGraph 2 [52]

*Pseudomonas veronii* strain R4 is a motile, Gram-negative, nonsporulating rod in the order *Pseudomonadales* of the class *Gammaproteobacteria*. The rod-shaped form varies in size with dimensions of 0.6 μm in width and 2.0 μm in length (Fig. 2a). It is fast growing, forming 2 mm diameter colonies after 48–72 h when grown on KB) [13] at 28 °C. Colonies on KB are white/yellow-opaque, slightly domed, and moderately mucoid with smooth margins (Fig. 2b). The strain R4 was isolated from the roots of healthy nursery-produced grapevine plants in the Maipo valley (Central Chile). It can grow in complex media such as LB [14] or KB as well as in minimal media such as M9 medium [15]. The optimal growth temperature is 28 °C; however, the strain R4 can still replicate at 5 °C in liquid LB and KB. Growth at 37 °C was not observed in these culturing media after 24 h. The bacterium is a colonizer of the grapevine rhizosphere, and it does not cause any deleterious effect on its original host. The strain R4 has natural resistance to carbenicillin (100 mg/L), cefotaxime (300 mg/L), and the mixture of ticarcillin:potassium clavulanate 15:1 (250 mg/L). Minimum Information about the Genome Sequence of *P. veronii* strain R4 is summarized in Table 1. A phylogenetic tree for the strain R4 and other *Pseudomonas* spp. was built using a concatenated alignment of 31 universal protein families (Additional file 1: Table S1, Fig. 3).

**Table 1:** Classification and general features of the *Pseudomonas veronii* strain R4 [39]

MIGS ID	Property	Term	Evidence code^a^
	Classification	Domain: *Bacteria*	TAS [40]
		Phylum: *Proteobacteria*	TAS [41]
		Class: *Gammaproteobacteria*	TAS [42]
		Order: *Pseudomonadales*	TAS [43]
		Family *Pseudomonadaceae*	TAS [44]
		Genus *Pseudomonas*	TAS [11]
		Species *Pseudomonas veronii*	TAS [12]
		strain: R4	
	Gram stain	Negative	TAS [45]
	Cell shape	Rod-shaped	TAS [45]
	Motility	Motile	TAS [45
	Sporulation	Not reported	NAS
	Temperature range	5-37 °C	TAS [12]
	Optimum temperature	28 °C	TAS [12]
	pH range; Optimum	neutral pH	TAS [12]
	Carbon source	Heterotrophic	TAS [12]
MIGS-6	Habitat	Soil, vine root-associated	TAS
MIGS-6.3	Salinity	0.85 % NaCl (w/v)	IDA
MIGS-22	Oxygen requirement	Aerobic	IDA
MIGS-15	Biotic relationship	Rizosphere	NAS
MIGS-14	Pathogenicity	Non-pathogen	IDA
MIGS-4	Geographic location	Chile/Los Andes Province	NAS
MIGS-5	Sample collection	2009	NAS
MIGS-4.1	Latitude	S 32° 50′ 42″	NAS
MIGS-4.2	Longitude	W 70° 36′ 57.599″	NAS
MIGS-4.4	Altitude	830 M	NAS

^a^Evidence codes - *IDA* Inferred from Direct Assay, *TAS* Traceable Author Statement (i.e., a direct report exists in the literature), *NAS* Non-traceable Author Statement (i.e., not directly observed for the living, isolated sample but based on a generally accepted property for the species or anecdotal evidence). These evidence codes are from the Gene Ontology project [46]

## Genome sequencing information

### Genome project history

*P. veronii* strain R4 was selected for sequencing due to the following: its environmental and agricultural potential; its ability to exert in vitro biocontrol against nematode *X. index;* and its ability to develop a symbiotic relationship with grapevine root tissues. The genome project is deposited in the Genomes OnLine Database, GOLD [16], and the NCBI BioProject database. The draft genome sequence is in GenBank. A summary of the project information is shown in Table 2.

**Table 2:** Project information

MIGS ID	Property	Term
MIGS 31	Finishing quality	High-quality Draft
MIGS-28	Libraries used	8,000 bp Mate Pair
MIGS 29	Sequencing platforms	454 GS-FLX Titanium
MIGS 31.2	Fold coverage	52.0
MIGS 30	Assemblers	GS De Novo Assembler V2.9
MIGS 32	Gene calling method	RAST 2.0, GLIMMER 3.0
	Locus Tag	SU91
	Genbank ID	JXWQ00000000
	GenBank Date of Release	April 22, 2015
	GOLD ID	Gp0114890
	BIOPROJECT	PRJNA272785
MIGS 13	Source Material Identifier	R4
	Project relevance	Biotechnological, Agricultural

### Growth conditions and genomic DNA preparation

A 2-mL overnight culture of strain R4 was prepared in a liquid KB medium at 28 °C and 150 rpm. Two hundred microliters from this culture were used as an inoculum for 200 mL of KB medium and incubated for an additional 8 h under the same culture conditions. The bacteria were centrifuged at 3000 × *g* and subjected to DNA purification using the ZR Fungal/Bacterial DNA MiniPrep™ kit (Zymo Research), according to the manufacturer’s protocol. The concentration and purity of DNA was measured by a BioSpec-Nano spectrophotometer (Shimadzu Corp., Kyoto, Japan). Five micrograms of purified genomic DNA were submitted for the 454 pyrosequencing.

### Genome sequencing and assembly

The genome of the strain R4 was sequenced at Macrogen (Macrogen Inc., Seoul, South Korea) using the 454 sequencing platform. The data consisted of a half plate of 454 FLX Titanium from 8 KB mate-paired libraries. A total of 794,931 reads were achieved for this characterization study, yielding 352,645,131 bases and an average read length of 443.618 bases. The GS De Novo Assembler 2.9 (also known as Newbler assembler) developed by 454 Life Sciences (Roche Company, Basel, Switzerland) was used for sequence assembly, quality assessment, and scaffolding.

### Genome annotation

The genes in the assembled genome were predicted with Rapid Annotation using Subsystem Technology server databases 2.0 [17] and the gene-caller GLIMMER 3.02 [18]. Clusters of Orthologous Groups of proteins functional classification was based on homology searches using WebMGA [19]. RNAmmer 1.2 [20] and tRNAscan-SE 1.4 [21] were used to identify rRNA genes and tRNA genes, respectively. CRISPR repeats were examined using the CRISPR recognition tool [22]. Signal peptides and transmembrane helices were predicted using SignalP [23] and TMHMM [24], respectively.

### Genome properties

**Fig. 4:**
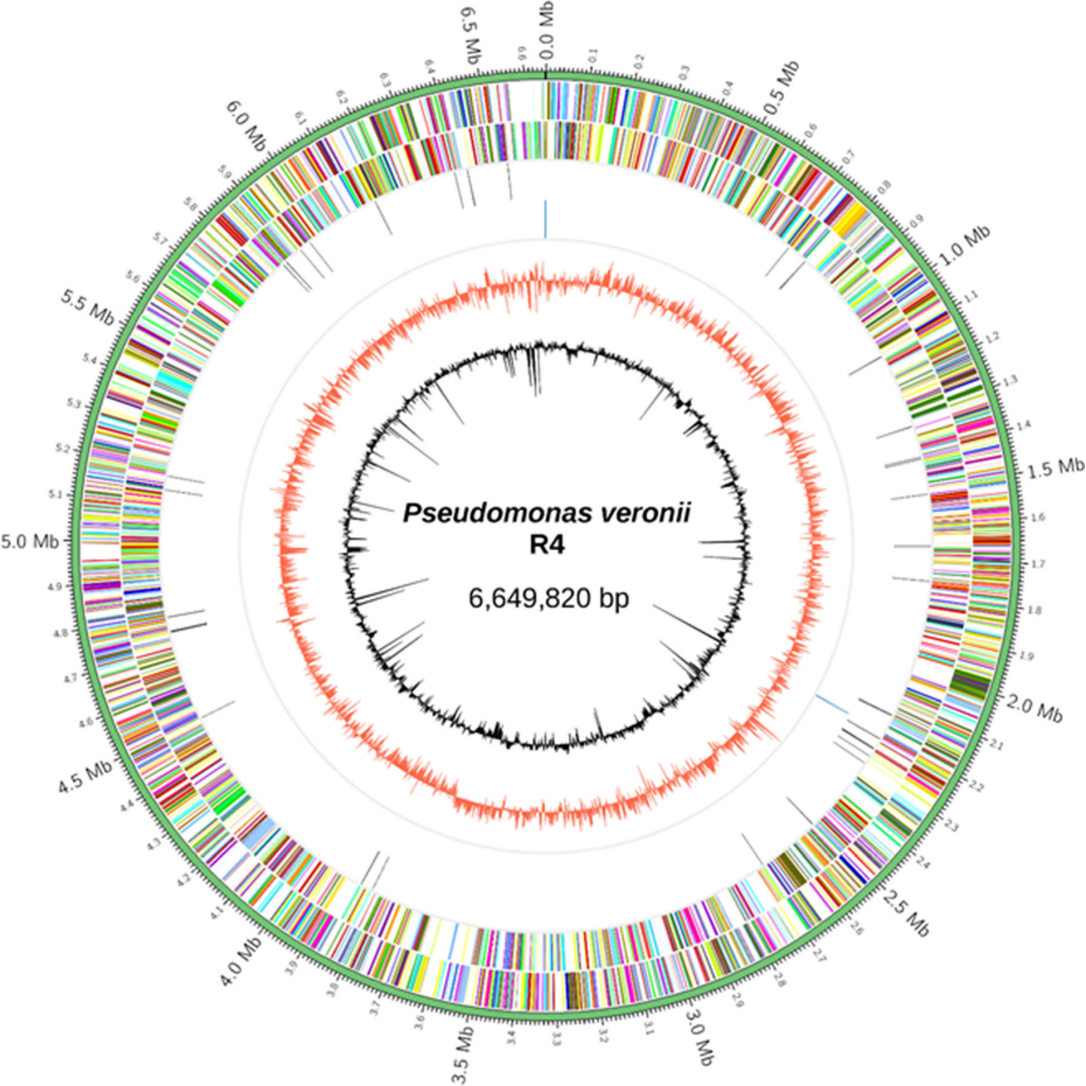
Graphical map of the chromosome. From outside to the centre: genes on forward strand (coloured by COG categories), genes on reverse strand (coloured by COG categories), RNA genes (tRNAs – black, rRNAs – *blue*, GC skew in *red*, and G + C in relation to the mean G + C – *black*), which are both in 2 kb windows. The circular map was generated by Circos [53]

The assembly of the draft genome sequence comprises two scaffolds amounting to 6,649,820 bp (60.8 % average GC content) and a N50 of 6,647,193. In total, 5967 genes were predicted (Table 3, Fig. 4), 5906 of which are protein-coding genes and 61 of which were RNA genes (3 rRNA genes and 58 tRNA genes). The majority of the protein-coding genes (82.9 %) were assigned to a putative function with the remaining annotated as hypothetical proteins. The distribution of genes into COGs functional categories is presented in Table 4.

**Table 3:** Genome statistics

Attribute	Value	% of Total
Genome size (bp)	6,649,820	100.0
DNA coding (bp)	5,735,337	86.2
DNA G + C (bp)	4,043,431	60.8
DNA scaffolds	2	–
Total genes	5,967	100.0
Protein coding genes	5,906	99.0
RNA genes	61	1.0
Pseudo genes	–	–
Genes in internal clusters	–	–
Genes with function prediction	4,894	82.9
Genes assigned to COGs	4,923	83.4
Genes with Pfam domains	4,799	80.4
Genes with signal peptides	557	9.4
Genes with transmembrane helices	1,322	22.4
CRISPR repeats	0	0

**Table 4:** Number of genes associated with general COG functional categories

Code	Value	% age	Description
J	190	3.22	Translation, ribosomal structure and biogenesis
A	1	0.02	RNA processing and modification
K	523	8.86	Transcription
L	155	2.62	Replication, recombination and repair
B	3	0.05	Chromatin structure and dynamics
D	45	0.76	Cell cycle control, Cell division, chromosome partitioning
V	81	1.37	Defense mechanisms
T	445	7.53	Signal transduction mechanisms
M	271	4.59	Cell wall/membrane biogenesis
N	211	3.57	Cell motility
U	157	2.66	Intracellular trafficking and secretion
O	190	3.22	Posttranslational modification, protein turnover, chaperones
C	340	5.76	Energy production and conversion
G	303	5.13	Carbohydrate transport and metabolism
E	569	9.63	Amino acid transport and metabolism
F	109	1.85	Nucleotide transport and metabolism
H	212	3.59	Coenzyme transport and metabolism
I	235	3.98	Lipid transport and metabolism
P	308	5.22	Inorganic ion transport and metabolism
Q	156	2.64	Secondary metabolites biosynthesis, transport and catabolism
R	611	10.35	General function prediction only
S	470	7.96	Function unknown
–	983	16.64	Not in COGs

The total is based on the total number of protein coding genes in the genome

## Insights from the genome sequence

*In-silico* DNA-DNA hybridization was performed using a Genome Blast Distance Phylogeny approach to generate genome based distance measures for phylogenetic inferences and also demonstrated the close relationship between the strain R4 and *Pseudomonas veronii*. The Genome-to-Genome Distance Calculator [25] revealed a distance of 0.0127 between strain R4 and *P. veronii* 1YdBTEX2, with a DDH estimate of 89.50 % +/− 2.16. A DDH similarity above 70 % is interpreted as two individuals belonging to the same species, and 79 % is used to discriminate between subspecies [26]. The DDH estimate exceeding the 70 % species threshold was determined from a logistic regression to be 95.63 %. In terms of subspecies relatedness, the probability of exceeding the 79 % threshold was 64.46 % between strain R4 and 1YdBTEX2.

### Typical *P. veronii* elements

Regular genetic elements associated with heavy metal tolerance are found in the strain R4, as expected for a *P. veronii* isolate. The *copABCD* operon encodes a multicopper oxidase CopA, which oxidizes cathecol siderophores and generates Cu^2+^ chelating pigments; in addition, copper-binding proteins (such as CopB, CopC, CopD) that decrease oxidative stress [27, 28] were also found in strain R4 and showed an identical organization to the *P. veronii* 1YdBTX genome [29] and depicted high identity of encoded proteins showing values >60 %. Also, the strain R4 possesses the gene cluster *cznCBA* and the *cznD* gene, encoding for a cation efflux pump of the Resistance-Nodulation-Division family and for a cationic diffusion enhancer, respectively. Both elements confer Zn^+2^ and Cd^+2^ resistance in C. metallidurans CH34 [30], and they can be identified in the *P. veronii* 1YDBTX genome [29].

Genetic elements associated with organic phosphate mineralization from soil were found as extracellular alkaline phosphatase genes *PhoD* and *PhoX*. These have been characterized in *Pseudomonas fluorescens* Pf-0 and allow for soluble phosphate generation and plant absorption [31]. The strain R4 also possesses the *amoA* gene, which encodes for ammonia mono-oxygenases involved in ammonia-to-nitrite transformation and increases nitrogen availability in soil [32]. Approximately 45 ORFs appeared involved in the denitrification process, which could be organized in three clusters with high homology and with the same organization as in *P. fluorescens* F113 [33]*.*

### Plant-microbe interaction elements

Different plant-bacteria interaction pathways including chemotaxis, adherence, root colonization, nutrients uptake, auxin synthesis, and volatile compound synthesis were deduced from the R4 genome. A two-component chemotaxis system including the kinase sensor (CheA) and the response regulator (CheY) plus potential plant exudate chemoreceptors could activate cell motility into roots, which could be mediated by the flagellar system conformed by almost 77 genes (see Table 5). Root colonization by strain R4 could then be possible by adhesin-like proteins (haemagglutinin and *pili* types) mediating the plant cell surface association and contact inhibition. Whereas haemagglutining-like genes are distributed throughout the entire genome, Type IV *pili* genes clustered into two-component systems, signal transduction, and *pili* structural gene clusters. Also, central carbohydrate metabolism (tricarboxylic acids, Entner-Doudoroff, and pentose cycles) suggests a broad carbon source usage (i.e., D-mannitol, sucrose, trehalose, maltose, xylose, and glucose) derived from plant exudates. They may also use some of the several transporter systems such as regulated by PTS that were also annotated. Supporting these ideas, several GABA catabolic enzymes were found in the strain R4, such as the *gadT* and *gadD* genes, which encode for the GABA aminotransferase and succinate semialdehyde dehydrogenase. GABA is a non-proteinogenic amino acid secreted by plants in order to inhibit herbivore, bacterial, and fungal pathogens. The GABA degradative products could be incorporated into the tricarboxylic acids cycle and provide additional carbon provision for the bacteria upon interaction with plants. In addition, the strain R4 presented genes for ABC, MFS, and RND family transporters, enabling nutrient exchange from and into the rhizosphere.

**Table 5:** Relevant gene clusters identified on the R4 genome associated with plant-microbe interactions

Module	Components	Associated genes	Function in strain R4
Tricarboxylic acids cycle	Catabolic genes	15	Carbon metabolism from plant exudates (mannitol, sorbitol, sucrose, trehalose, mannose, arabinose, maltose, xylose and glucose)
Entner-Doudoroff pathway	Catabolic genes	5	
Pentose cycle	Catabolic genes	14	
Rizosphere nutrients uptake	ABC tranporters regulated by PTS	23	
Chemotaxis	CheA, CheB, CheR, CheW, CheY and chemoreceptors	70	Sensing chemical stimulus and direct motility
Motility	Flagello: estructural genes and regulatory genes	85	Motility
Root colonization	Type IV pili: estructural genes and two components signal transduction proteins	24	Host cell surface association and host growth inhibition by contact
	Haemagglutinin genes	4	
	Alginate: biosinthetic and regulatoy genes	24	Biofilms
Transporters	MFS genes	32	Transporters involved in bacterium - rizosphere interaction
	RND genes	43	
	ABC genes	162	
Acetoin and 2,3-butanediol synthesis	ilvBN; budC; bdh; acoABCX adh	10	Plant growth regulators synthesis and catabolism
IAA	Two synthetic pathways: from indol-3-acetamide and from indole-3-acetonitrile	9	
Ethylene	acdS	1	ACC catabolism
GABA	gadT, gadD, GABA permease gene	5	c-aminobutiric acid synthesis
Proteases and lipases	Exportable protease (AprA), lipase (LipA) and phopholipase ExoU-like	8	biocontrol activity
Secondary metabolites	Pyoverdine: estructural genes, and regulatory genes	19	
	Pyochelin: estructural genes and regulatory genes	30	
Secretion systems	Type I; Type II; TypeIII and Type VI	85	Transport of biocontrol molecules

Under in vitro conditions, the strain R4 was found to produce IAA from tryptophan; this behaviour was markedly different from other rhizobacteria such as *Rhizobium* spp. 13, in which differential IAA accumulation has been observed depending on the precursor concentration [34]. The genome in strain R4 contained two potential IAA synthetic pathways from tryptophan: a) the indole-3-acetamide and b) the indole-3-acetonitrile synthetic routes (Table 5). In addition, the R4 genome presents complete synthetic routes for acetoine biosynthesis, i.e., the *ilv*BN and the acetoine reductase (*budC*) genes and also the synthetic *bdh* and the catabolic *acoABCX adh* genes [10, 35]. These results suggest that strain R4 could transform acetoine into 2,3-butanediol and maybe other PGPs. In addition, the genome data analysis showed the occurrence of a complete catabolic pathway for ethylene, a root elongation inhibitor. The occurrence of the carboxylate-1-aminocyclopropane deaminase (*acdS*) gene could potentially degrade the ethylene precursor aminocyclopropane into ketobutyric and ammonia, which could synergize the indicated PGP activities.

### Biocontrol elements

A gene cluster of 11 Kb in length conserved in *P. fluorescens* strains SBW25, A506, SS101, F113 [10, 33] and strain R4 included two secretories enzymes, one protease (similar to the metalloprotease Apra very relevant in CHA0 [7]), and one lipase plus an ABC transporter involved in proteases secretion [36]. In CHA0, AprA has been described to inhibit *Meloidogyne incognita* egg hatching and the death of young nematode individuals [7]. Three other potential exoproteases and two exolipases that have not been described in *P. fluorescens* were found. A phospholipase (68 kDa) similar in size to the *P. aeruginosa* ExoU (Acc. N^o^ ABJ10150.1) protein, was annotated in the R4 genome. The latter corresponds to an effector protein of the Type III Secretory System in *P. aeruginosa*, one of the most important virulence factor in that species [37, 38].

## Conclusions

The genome analysis allowed for the identification of gene clusters encoding for putative extracellular proteases, lipases, and eventual transport systems that are proposed to mediate, at least in part, the nematicidal activity found in this *P. veronii* strain in a *X. index* biocontrol panel. In addition, bioinformatics analyses supported preliminary experimental data that describe plant growth promotion through a putative IAA synthesis pathway.

The phylogenetic relationships between the strain R4 and other sequenced *Pseudomonas* spp. strains on the basis of concatenated alignment of 31 universal protein families showed the closest relationship with *P. veronii* strains 1YdBTEX2 and *P. extremaustralis* 14–3 sbstr. 14-3b. These formed a clade with a high similarity to a group conformed by numerous *P. fluorescens* isolates.

A predicted R4 genome consisted of 6,678,155 bases in which an assignment of 5840 CDS depicted a coding density of 86.8 %. Using a functional classification of 3796 CDS (65 % of total CDS) by comparing protein sequences from complete genomes and executing a COG, candidate gene sequences revealed several functions such as complete pathways related to carbohydrate central metabolism (i.e., the tricarboxylic acid cycle, the Entner-Doudoroff pathway, and the pentose cycle), and metabolic routes related to plant–bacteria interactions were found. Similarly, metabolic pathways for the synthesis of PGPs such as IAA, acetoin, and 2,3-butanediol were also successfully identified. Moreover, gene groups for chemotaxis, root colonization, rhizosphere nutrient uptake, and volatile compounds were found.

## Additional file

Additional file 1:
**Table S1.**Cluster of Orthologous Genes (COG) considered in the phylogenetic analysis. (DOCX 12 kb)

## Abbreviations

2,4 DAPG: 2,4-diacetylphloroglucinol
DDH: Digital DNA-DNA hybridization
GABA: Gamma-aminobutiric acid
IAA: Indole acetic acid
KB: King’s agar
PGPs: Plant growth promoters
PTS: Phosphotransferase system
