# Supplementary material for: A draft genome sequence of Pseudomonas veronii R4: a grapevine (Vitis vinifera L.) root-associated strain with high biocontrol potential
Source: Stand Genomic Sci. 2016 Oct 11;11:76. doi: 10.1186/s40793-016-0198-y (PMC5057446; doi:10.1186/s40793-016-0198-y)
Supplement: Additional file 1: — Table S1.Cluster of Orthologous Genes (COG) considered in the phylogenetic analysis. (DOCX 12 kb) [file 40793_2016_198_MOESM1_ESM.docx]

**Supplementary Table S1.** Cluster of Orthologous Genes (COG) considered in the phylogenetic analysis.

| COG | Description |  |
| --- | --- | --- |
| COG0012 | GTP-binding protein YchF |  |
| COG0016 | Phenylalanyl-tRNA synthetase alpha subunit |  |
| COG0048 | Ribosomal protein S12 |  |
| COG0049 | Ribosomal protein S7 |  |
| COG0052 | 30S ribosomal protein S2 |  |
| COG0080 | 50S ribosomal protein L11 |  |
| COG0081 | 50S ribosomal protein L1 |  |
| COG0087 | 50S ribosomal protein L3 |  |
| COG0091 | 50S ribosomal protein L22 |  |
| COG0092 | 30S ribosomal protein S3 |  |
| COG0093 | Ribosomal protein L14 |  |
| COG0094 | Ribosomal protein L5 |  |
| COG0096 | 30S ribosomal protein S8 |  |
| COG0097 | 50S ribosomal protein L6 |  |
| COG0098 | Ribosomal protein S5 |  |
| COG0099 | Ribosomal protein S13 |  |
| COG0100 | Ribosomal protein S11 |  |
| COG0102 | 50S ribosomal protein L13 |  |
| COG0103 | Ribosomal protein S9 |  |
| COG0172 | seryl-tRNA synthetase |  |
| COG0184 | 30S ribosomal protein S15 |  |
| COG0186 | Ribosomal protein S17 |  |
| COG0197 | 50S ribosomal protein L16 |  |
| COG0200 | 50S ribosomal protein L15 |  |
| COG0201 | Preprotein translocase subunit SecY |  |
| COG0202 | DNA-directed RNA polymerase subunit alpha |  |
| COG0256 | Ribosomal protein L18 |  |
| COG0495 | Leucyl-tRNA synthetase |  |
| COG0522 | Ribosomal protein S4 |  |
| COG0525 | Valyl-tRNA synthetase |  |
| COG0533 | UGMP family protein |  |
